# Micro-Evolution Analysis Reveals Diverged Patterns of Polyol Transporters in Seven Gramineae Crops

**DOI:** 10.3389/fgene.2020.00565

**Published:** 2020-06-19

**Authors:** Weilong Kong, Tong Sun, Chenhao Zhang, Yalin Qiang, Yangsheng Li

**Affiliations:** State Key Laboratory of Hybrid Rice, College of Life Sciences, Wuhan University, Wuhan, China

**Keywords:** polyol transporters, Gramineae crops, orthologous gene pairs, orthogroups, duplicate gene pairs, functional differentiation

## Abstract

Polyol transporters (PLTs), also called polyol/monosaccharide transporters, is of significance in determining plant development and sugar transportation. However, the diverged evolutionary patterns of the *PLT* gene family in Gramineae crops are still unclear. Here a micro-evolution analysis was performed among the seven Gramineae representative crops using whole-genome sequences, i.e., *Brachypodium distachyon* (Bd), *Hordeum vulgare* (Hv), *Oryza rufipogon* (Or), *Oryza sativa* (Os), *Sorghum bicolor* (Sb), *Setaria italica* (Si), and *Zea mays* (Zm), leading to the identification of 12, 11, 12, 15, 20, 24, and 20 *PLT* genes, respectively. In this study, all *PLT* genes were divided into nine orthogroups (OGs). However, the number of *PLT* genes and the distribution of *PLT* OGs were not the same in these seven Gramineae species, and different OGs were also subject to different purification selection pressures. These results indicated that the *PLT* OGs of the *PLT* gene family have been expanded or lost unevenly in all tested species. Then, our results of gene duplication events confirmed that gene duplication events promoted the expansion of the *PLT* gene family in some Gramineous plants, namely, Bd, Or, Os, Si, Sb, and Zm, but the degree of gene family expansion, the type of *PLT* gene duplication, and the differentiation time of duplicate gene pairs varied greatly among these species. In addition, the sequence alignment and the internal repeat analysis of all PLTs protein sequences implied that the PLT protein sequences may originate from an internal repeat duplication of an ancestral six transmembrane helical units. Besides that, the protein motifs result highlighted that the PLT protein sequences were highly conserved, whereas the functional differentiation of the *PLT* genes was characterized by different gene structures, upstream elements, as well as co-expression analysis. The gene expression analysis of rice and maize showed that the *PLT* genes have a wide range of expression patterns, suggesting diverse biological functions. Taken together, our finding provided a perspective on the evolution differences and the functional characterizations of *PLT* genes in Gramineae representative crops.

## Introduction

Sugar is the main product of plant photosynthesis. Sugar (monosaccharides, sucrose, and polyols) plays an important role in the entire life cycle of plants (Deng et al., 2019; Kong et al., 2019a). It can not only provide energy for the growth and the development of plants but also is used for storage and transportation (Kühn and Grof, 2010). In various plant metabolic pathways, sugar can be used as a signal molecule (Wingenter et al., 2010; Kong et al., 2019a). Plant sugar transporters mainly mediate the transportation of sugars, participate in the loading and unloading of various sugars between “source and sink” tissues, and affect the distribution of plant carbohydrates (Kong et al., 2019a; Misra et al., 2019; Patil et al., 2019). To date, more and more sugar transporters have been identified and experimentally verified in various plant species, namely, monosaccharide transporters (MSTs), sucrose transporters, and sugars will eventually be exported transporters (Jeena et al., 2019; Kong et al., 2019a; Misra et al., 2019).

As we all know, polyol transporters (PLTs) belong to a subfamily of the MST superfamily (Deng et al., 2019; Kong et al., 2019a). Previous studies have demonstrated that PLTs can transport polyols and monosaccharides, also known as polyol/monosaccharide transporters (PMTs) (Büttner, 2007; Klepek et al., 2010). Besides that, PLTs reportedly loaded polyols into the phloem or unloaded sugars from the phloem for accumulation in sink tissues (Noiraud et al., 2001; Watari et al., 2004; Conde et al., 2007; Juchaux-Cachau et al., 2007). The first PLT was identified in celery (*Apium graveolens*), named AgMaT1, which had a high affinity for mannitol (Km = 275 μM) and showed a high expression level in mature leaves (Noiraud et al., 2001). Next, AgMaT2 was identified as an H(+)/mannitol co-transporter, which was less selective for other polyol molecules in celery and played a role in defense mechanisms (Juchaux-Cachau et al., 2007). What is more, Arabidopsis PMT5 (AtPMT5) transported a broad range of sugars, including cyclic and linear polyols, hexoses, and pentoses (Klepek, 2005; Reinders et al., 2005; Klepek et al., 2010). A tissue expression analysis showed that *AtPMT1* and *AtPMT2* were relatively highly expressed in some immature tissues, such as young germinating pollen and pollen tubes. Notably, they had a high ability to transport fructose (Klepek, 2005; Klepek et al., 2010). In *Lotus japonicus*, 14 putative *PLT* genes were identified and three genes (*LjPLT1*, *LjPLT3*, and *LjPLT9*) among them were found to respond to salinity and/or osmotic stresses (Lu et al., 2017). Another example is the *VvPLT1* gene in grapevine, which was up-regulated under salt- and water-deficit stresses, as well as exogenous salicylic acid and abscisic acid treatments, and had a high affinity for sorbitol and mannitol (Conde et al., 2015). Also, *OeMaT1* in *Olea europaea* was significantly induced by salinity and drought stresses (Conde et al., 2011). Another study reported that *Hevea brasiliensis HbPLT2* expressing yeast displayed active absorption of xylitol but a marginal level of absorption of inositol, mannitol, and sorbitol (Dusotoit-Coucaud et al., 2010).

Previous studies have focused on the transport substrate, tissue-specific expression, and subcellular localization of PLTs (Kühn and Grof, 2010). However, the evolutionary dynamics of *PLT* genes in plants are still unclear. Given the strong transportation capacity of PLTs, they are of great significance to the growth and the yield increase of Gramineae crops. On the other hand, several Gramineae crops with high-quality genomes are ideal model systems to study the short-term evolutionary dynamics of gene families and their consequences (Kong et al., 2019a,b,c). Here seven Gramineae crops, namely, *Brachypodium distachyon* (Bd), *Hordeum vulgare* (Hv), *Setaria italica* (Si), *Sorghum bicolor* (Sb), *Zea mays* (Zm), *Oryza rufipogon* (Or), and *Oryza sativa* (Os), were selected for a systematical study of Gramineae *PLT* gene family, including evolutionary analysis, topology, 3D structure, genetic structure, conserved domains, gene duplication events, selection pressures, orthogroups (OGs), co-expression analysis, upstream elements in promoters, and tissue expression analysis. Our study will facilitate further molecular evolution and function study of Gramineae PLTs.

## Materials and Methods

### Plant Materials and Treatments

Seedlings of rice cultivar “9311” (*O. sativa* ssp. *indica*) were grown in containers with sponges as supporting materials in Yoshida solution in a Wuhan University greenhouse, with 16/8-h light/dark photoperiod at 26°C and 60% relative humidity (Deng et al., 2019; Kong et al., 2019a). The 12-day-old seedlings were changed to 40 and 4°C for the hot and the cold treatments. The leaves were collected at 0, 1, 3, 6, and 12 h after the hot or the cold treatment (Kong et al., 2019a).

In this study, three biological replicates of every treatment were generated, and each biological replicate was collected and pooled together from over 12 plants. To ensure the accuracy of the results, three technical replicates were set for each biological replicate in quantitative real-time PCR (qRT-PCR) (Kong et al., 2019d).

### Identification and Phylogenetic Tree Construction of PLT Genes

Firstly, the genome databases of Bd, Hv, Si, Sb, Zm, and Or were obtained from Ensembl Plants release 41^1^. A genome database of Os (MSU 7.0) was downloaded from TIGR Database^2^. The hidden Markov model profile of the Sugar_tr (PF00083) was downloaded from the Pfam website^3^. Then, all these candidate PTL protein sequences were separately obtained by HMMER 3.2.1 with default parameters (Finn et al., 2011) and BlastP homology search with E-value cutoff e^–5^ (Kong et al., 2017; Wu et al., 2019). Only the longest splice variant of each gene locus was adopted in this study. After removing the redundant sequences, all candidate PLT protein sequences were submitted to SMART^4^, Pfam^5^, and National Center for Biotechnology Information Protein Basic Local Alignment Search Tool^6^ to confirm the highly conserved Sugar_tr domain (Kong et al., 2017; Kong et al., 2019d).

Multiple sequence alignment of all full-length PLT protein sequences was performed using MUSCLE with default parameters (Edgar, 2004). We used ModelFinder software to determine that the best substitution model of the aligned PLT protein sequences was JTT + F + G4 (Kalyaanamoorthy et al., 2017). Subsequently, the tree reconstruction was conducted by the IQ-tree software with a bootstrap test for 1,000 replicates and an SH-aLRT test for 1,000 random addition replicates (Nguyen et al., 2015).

### Orthogroup Analysis of PLTs, Gene Duplicate Events, and Selective Stress Analysis

*PLT* OGs were identified using OrthoFinder software with default parameters after an all-*vs*-all BlastP search of PLT protein sequences with E-value of 1 e^–3^ (Emms and Kelly, 2015; Kong et al., 2019d). Additionally, the selective forces on OGs were evaluated by Tajima’s *D*-values using DnaSP 5.0 (Librado and Rozas, 2009; Kalyaanamoorthy et al., 2017).

In order to explore the expansion mechanism of the *PLT* gene family in Gramineous crops, the gene duplication events of *PLT* genes were analyzed with default parameters using the “duplicate_gene_classifier” script in Multiple Collinearity Scan toolkit X version (MCScanX) after a BlastP search of intraspecies PLT protein sequences with an E-value of 1 e^–5^ (Wang et al., 2012; Kong et al., 2019a). Next, the physical gene locations and the gene duplication events of *PLT* genes were visualized by Circos (Krzywinski et al., 2009).

In this study, we used DnaSP v5.0 software to determine non-synonymous (Ka) to synonymous (Ks) substitution rates (Ka/Ks) of duplicate gene pairs in seven tested species (Librado and Rozas, 2009; Kong et al., 2019b). Furthermore, the divergence time of the duplicate gene pairs was predicted using the formula: *T* = Ks/(2 × 9.1 × 10^–9^) × 10^–6^ Mya (Deng et al., 2019; Kong et al., 2019c).

### Internal Repeat Analyses, Transmembrane Helical Domains, 3D Structure Analysis, Conserved Motifs, and Gene Structures

The multiple alignment result of all PLT protein sequences was generated by the Clustal X software (Thompson et al., 1997) for internal repeat analyses for exploring the generation mechanism of PLT proteins. HHrepID was used to generate the internal repeats with these parameters: maximal number of multiple sequence alignment generation steps was one, score secondary structure was no, repeat family *P*-value threshold was 1 e^–2^, self-alignment *P*-value threshold was 1 e^–1^, merge rounds was three, and domain boundary detection was no (Biegert and Söding, 2008). At the same time, we also predicted the transmembrane helical (TMH) domains of all PLT protein alignment results in TMHMM Severv.2.0^7^ (Kong et al., 2019b). On the other hand, the 3D structure models of all rice PLT protein sequences were analyzed in the SWISS-MODEL website^8^ with default parameters, and the best template result was chosen for each PLT protein sequence (Waterhouse et al., 2018).

The MEME online tool^9^ was used to search the conserved motifs of all PLTs protein sequences, with a motif width of 6–100 amino acids, a limit of 20 motifs, and all other default parameters (Bailey et al., 2015). A gene exon–intron structure analysis of all *PLT* genes was performed on the Gene Structure Display Server 2.0 (GSDS 2.0^10^) (Hu et al., 2015).

### Quantitative Analysis of Rice *PLT* Genes Under Heat and Cold Stresses

All RNAs were extracted by the TRIzol reagent (Invitrogen, Beijing, China) and reversed to cDNAs using HiScript III 1st Strand cDNA Synthesis Kit (Vazyme, Shanghai, China). The qRT-PCR reactions (10 μl) were formulated using 2× SYBR Green qPCR Master Mix (US Everbright^®^ Inc., Suzhou, China), following the manufacturer’s instructions. The qRT-PCR reactions were detected on a CFX96 Touch^TM^ Real-Time PCR Detection System (Bio-Rad, Hercules, CA, United States). The actin gene was used as an internal control (An et al., 2017; Kong et al., 2019b), and relative expression values were calculated by 2^–ΔΔCT^ method based on three biological replicates, with each replicate having three technical replicates (Kong et al., 2019d).

### Cis-Elements, Expression Analyses, and Co-expression Analysis

Two-kbp upstream sequences from the transcription start site of all *PLT* genes in these seven tested species were regarded as promoter sequences and extracted from gff3 and genetic fasta using Gtf/Gff3 sequences extractor tool in TBtools (Chen et al., 2018). Then, all upstream promoter sequences were submitted to the PLANTCARE website for the cis-elements identification (Lescot et al., 2002). To determine the expression patterns of *PLT* genes in rice and maize, the RNA-seq data of various tissues from rize and maize were obtained from the MBKbase database^11^ (Peng et al., 2020) and the maize eFP Browser^12^ (data source: Hoopes et al., 2019), respectively. The fragments per kilobase of transcript per million mapped reads values were used to represent the gene expression levels in this study, and all heatmaps were made by R package (pheatmap) (Kolde, 2012).

To understand the potential molecular regulation and gene network, the correlation coefficient between *PLT* genes was calculated based on Pearson coefficient. On the other hand, the co-expression network of rice *PLT* genes was constructed based on massive expression data (including 9 tissues, 24 projects, and 284 experiments) using a co-expression tool in the Rice Expression Database (RED^13^). Here the MSU 7.0 gene IDs of the *PTL* genes were input IDs, and the Pearson’s *r*-value was 0.85. The co-expressed gene network was visualized with Cytoscape V 3.6.1 (Shannon et al., 2003). Next, a gene ontology (GO) enrichment analysis of the obtained co-expressed genes was implemented by the GOseq R packages based on Wallenius non-central hyper-geometric distribution (Young et al., 2010).

## Results

### Identification and OG Classification of *PLT* Genes

**FIGURE 1:**
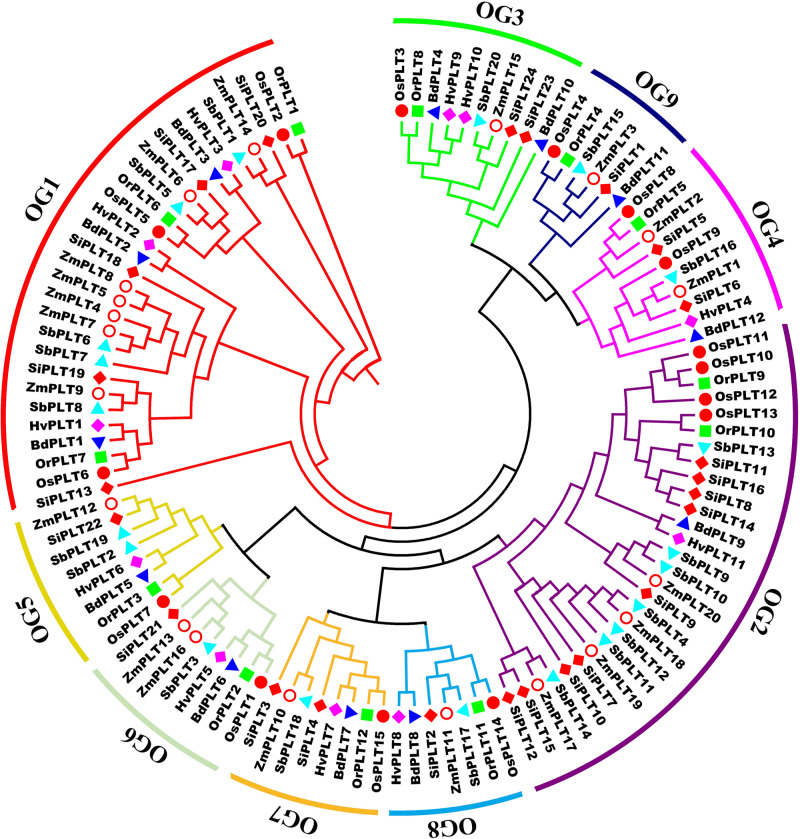
A maximum likelihood phylogeny tree of the polyol transporter protein sequences from seven Gramineae crops, namely, *Brachypodium distachyon* (Bd), *Hordeum vulgare* (Hv), *Setaria italica* (Si), *Sorghum bicolor* (Sb), *Zea mays* (Zm), *Oryza rufipogon* (Or), and *Oryza sativa* ssp. *japonica* (Os). This tree was established by IQ-tree software with a 1,000-replicate bootstrap test and a 1,000-random-addition-replicate SH-aLRT test. The colors in the outside circles and the inside branches represent different orthogroups (OG), including OG1, OG2, OG3, OG4, OG5, OG6, OG7, OG8, and OG9. Markers of different shapes markers indicate the different species in the phylogeny tree. The orthogroup analysis was performed using OrthoFinder v2.0 software with default parameters.

**FIGURE 2:**
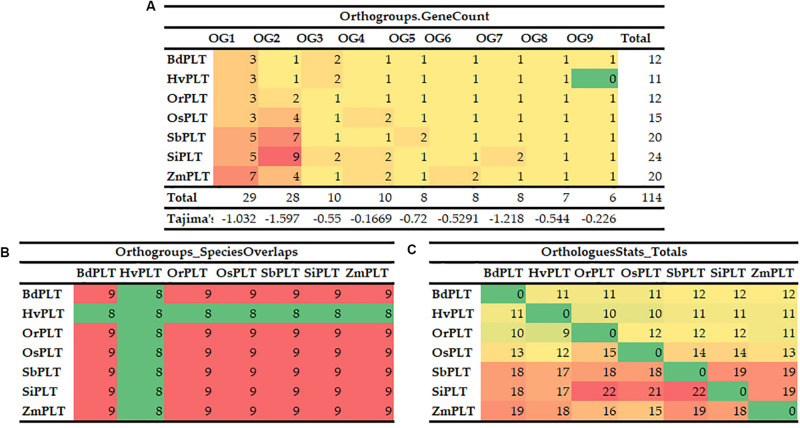
Orthogroups of *PLT* genes among the seven Gramineae crops, namely, BdPLT, HvPLT, OrPLT, OsPLT, SbPLT, SiPLT, and ZmPLT. Orthogroup overlaps **(A)**, statistical results of orthologous genes between different crops **(B)**, and gene counts and Tajima’s values of different orthogroups in these seven crops **(C)**.

A total of 114 *PLT* genes were identified in these seven tested Gramineae crops, namely, 12 in Bd, 11 in Hv, 12 in Or, 15 in Os, 20 in Sb, 24 in Si, and 20 in Zm (Figures 1, 2A, 5A and Supplementary Table S1). We found that Sb, Si, and Zm had more *PLT* genes than Bd, Hv, Or, and Os (Figures 1, 2C). In order to further analyze the reasons for the quantitative differences of the *PLT* genes in these tested species, a phylogenetic tree and OGs classification were conducted. As a result, all PLT proteins were clearly divided into nine OGs based on OrthoFinder analysis, namely, OG1, OG2, OG3, OG4, OG5, OG6, OG7, OG8, and OG9 (Figures 1, 2). The *PTL* genes in the same OGs were clustered together in the phylogenetic tree (Figures 1, 2 and Supplementary Tables S1, S2). We thus inferred that the number difference of the *PLT* gene among these tested species may be caused by the unequal loss and expansion of OGs in these different Gramineae crops (Figure 2 and Supplementary Table S2). For example, Hv had the smallest number of *PLT* genes, probably due to its loss of OG9 compared to other species in this study (Figure 2 and Supplementary Table S2). Another reason was that OG1 and OG2 expanded more obviously in Sb, Si, and Zm than in Bd, Hv, Or, and Os, which resulted in more *PLT* genes in Sb, Si, and Zm than in Bd, Hv, Or, and Os (Figure 2A and Supplementary Table S2). In addition, the specific expansion of OGs in the individual tested species also contributed to the difference in the number of *PLT* genes between species, namely, the specific expansion of OG5 in Sb, OG6 in Zm, and OG7 in Si (Figure 2A and Supplementary Table S2). Interestingly, OG8 was a single OG, which implied that the *PLT* gene in OG8 was very conservative during the evolution of Gramineae crops (Figure 2C and Supplementary Table S2). Furthermore, the orthologs were counted and summarized based on the OrthoFinder analysis result (Figure 2C and Supplementary Tables S3, S4). As expected, Sb, Si, and Zm had more orthologs than Bd, Hv, Or, and Os (Figure 2B). All orthologs could be divided into four types: many to many, one to one, many to one, and one to many (Supplementary Tables S3, S4). Finally, we evaluated the selection pressure of these nine OGs according to Tajima’s *D*-values using DnaSP 5.0 software. The result showed that the Tajima’s *D*-values of these nine OGs were less than 0, and from −0.1669 to −1.597 (Figure 2C), which denoted that these nine OGs were subjected to different intensities of purification selection pressure.

### Gene Duplicate Events of OGs and Selective Pressure of Duplicate Gene Pairs

**FIGURE 3:**
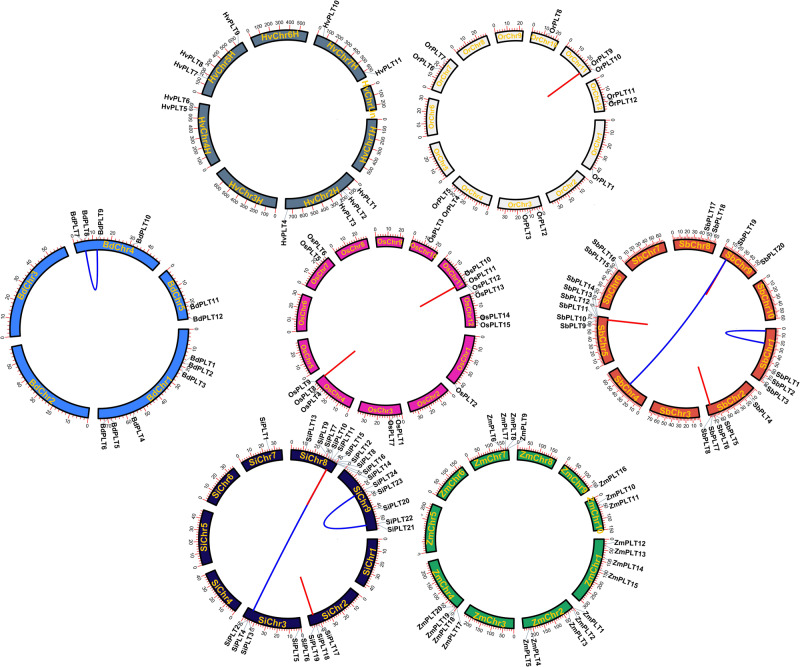
The chromosome locations and gene duplication events of *PLT* genes in seven species, namely, *Brachypodium distachyon* (Bd), *Hordeum vulgare* (Hv), *Setaria italica* (Si), *Sorghum bicolor* (Sb), *Zea mays* (Zm), *Oryza rufipogon* (Or), and *Oryza sativa* ssp. *japonica* (Os). The red line meant tandem duplication and the blue line meant whole-genome duplication/segmental duplication and tandem duplication.

In order to learn the specific expansion mechanism of OGs, duplicate gene pairs were identified and classified by MCScanX software (Wang et al., 2012). The result showed that a total of nine duplicate gene pairs originated from tandem duplication events, namely, one in Or, two in Os, two in Si, three in Sb, one in Zm, and zero in Hv and Bd (Figure 3 and Table 1). Besides that, six duplicate gene pairs from whole-genome duplication (WGD) or segmental duplication (SD) events were discovered in Bd (one), Zm (two), and Si (three). These results proved that gene duplications played significant roles in the OG expansion of the *PLT* gene family in these important Gramineae crops. Subsequently, the selective stress and the divergence time of these duplicate gene pairs were calculated to reveal the expansion differences of the *PLT* genes in these Gramineae crops. All Ka/Ks values of *PLT* duplicate gene pairs ranged from 0.25 to 0.77, suggesting that these duplicate gene pairs were mainly subject to purification selection pressure. Notably, the divergence times of the tandem duplication gene pairs were both later (<23 MYa) than that of the WGD/SD gene pairs (>24 MYa). These results indicated that the earlier expansion mode of all the Gramineous tested crops was WGD/SD and the later expansion mode was TD.

**TABLE 1:** Ka/Ks values and the divergence times of duplicate gene pairs in seven tested species.

**Seq_1**	**Seq_2**	**Orthogroup**	**Duplication type**	**Ka**	**Ks**	**Ka_Ks**	**The divergence time (Mya)**
SiPLT15	SiPLT12	OG2	TD	0.0407	0.11324	0.35943	6.2219856
OsPLT11	OsPLT12	OG2	TD	0.0777	0.15527	0.50042	8.5314223
SbPLT9	SbPLT10	OG2	TD	0.04197	0.16748	0.25057	9.2021418
SbPLT11	SbPLT12	OG2	TD	0.06961	0.19956	0.34881	10.964933
ZmPLT7	ZmPLT8	OG1	TD	0.06642	0.22753	0.2919	12.501534
OrPLT9	OrPLT10	OG2	TD	0.21296	0.31901	0.66758	17.528014
SbPLT6	SbPLT7	OG1	TD	0.08471	0.31966	0.265	17.56382
OsPLT8	OsPLT9	OG4	TD	0.1421	0.3455	0.4113	18.983323
SiPLT18	SiPLT19	OG1	TD	0.12165	0.40981	0.29684	22.517235
ZmPLT12	ZmPLT16	OG5	WGD/SD	0.20089	0.43838	0.45825	24.086673
ZmPLT4	ZmPLT6	OG1	WGD/SD	0.15838	0.45722	0.34639	25.122193
SiPLT2	SiPLT11	OG8	WGD/SD	0.41746	0.5403	0.77264	29.687065
BdPLT7	BdPLT9	OG7	WGD/SD	0.43375	0.60859	0.71272	33.439094
SiPLT3	SiPLT8	OG7	WGD/SD	0.37861	0.62256	0.60815	34.206506
SiPLT24	SiPLT22	OG3	WGD/SD	0.39954	0.70733	0.56485	38.864047

### TMH Prediction, Internal Repeat Analyses, Conserved Motifs, and Gene Structure Analysis

**FIGURE 4:**
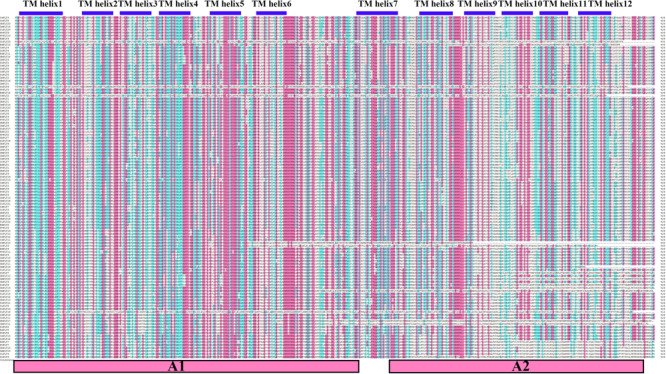
Sequence alignment result of polyol transporter protein sequences. The residues in red background represent the highly conserved residues. Twelve transmembrane helices were marked by blue boxes and the two functional duplicated regions are shown in red boxes at the bottom, namely, A1 and A2.

Our results of the protein sequence alignment and the TMH prediction revealed that all PLT protein sequences were strictly conserved and both had 12 TMHs, which supported the previous conclusion that PLT proteins consisted of 12 TMHs (Figure 4; Johnson et al., 2006). Interestingly, we also noticed that the first six TMHs were very similar to the next six TMHs (Figure 4). Therefore, we used the HHrepID program to perform the internal repeat analysis for all PLT protein sequences. As expected, we found that TMH1–TMH6 and TMH7–TMH12 were distributed in two duplicated regions, i.e., A1 and A2 (Figure 4 and Supplementary Figure S1), which implied that PLT protein sequences may originate from an internal repeat duplication of an ancestral six-TMH unit. Our findings were in agreement with the original hypotheses and provided a theoretical basis (Saier, 2000; Johnson et al., 2006). What is more, the 3D-structure models and ligands were predicted by the SWISS-MODEL website. We found that all PLT proteins belonged to two templates, namely, OsPLT4, OsPLT5, OsPLT6, OsPLT10, and OsPLT14 in 6h7d.1. A (description: sugar transport protein 10) and OsPLT1, OsPLT2, OsPLT3, OsPLT7, OsPLT8, OsPLT9, OsPLT11, OsPLT12, OsPLT13, and OsPLT15 in 6n3i.1. A (description: D-xylose transporter) (Supplementary Figure S2 and Supplementary Table S5). Among them, OsPLT4 had a ligand; the ligand was 1× OLC: (2R)-2,3-dihydroxypropyl(9Z)-octadec-9-enoate (Supplementary Table S5).

**FIGURE 5:**
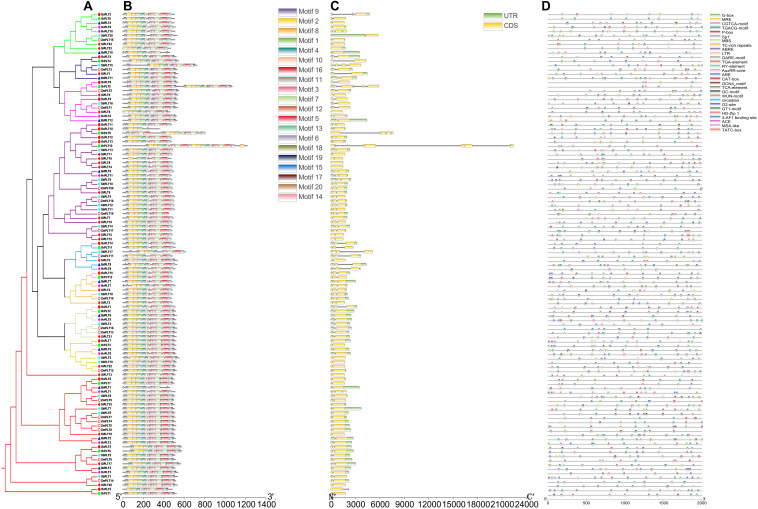
The phylogenetic tree **(A)**, conserved motif **(B)**, exon/intron compositions **(C)** and cis-elements **(D)** in the upstream promoters of the *PLT* genes in these seven species. The different colors of the branches represent different orthogroups and the different shapes of the markers mean different species in the phylogeny tree. The relative lengths of genes and proteins are shown by the width of the gray bars. The different motifs/cis-elements are distinguished by different colored boxes. The exons and introns are displayed by yellow boxes and gray lines, respectively.

To further explore the conservation and the sequence variation of the *PLT* genes in Gramineous crops, we separately utilized the MEME suite and GSDS 2.0 to predict the distribution of conserved domains and the intron–exon composition of all the *PLT* genes. Interestingly, all PLT protein sequences showed almost the same motif distribution, suggesting that the PLT proteins were very conserved in all tested species (Figure 5B). However, the *PLT* genes from the same OGs showed different intron–exon compositions among these seven tested species (Figure 5C). It was speculated that genetic sequence loss and exon fusion may occur during the evolution of the *PLT* gene family.

### Cis-Elements in Upstream Promoters and Gene Expression Analysis of Rice and Maize *PLT* Genes

The analysis of upstream elements and gene expression can provide a perspective for gene function speculation and functional differentiation research (Cao et al., 2019; Kong et al., 2019a; Zhang et al., 2020). We thus investigated the upstream elements of all the *PLT* genes in Gramineous crops and the *PLT* gene expression in various tissues of rice and maize at different developmental stages. Additionally, the expression changes of rice *PLT* genes was also detected by qRT-PCR.

**FIGURE 6:**
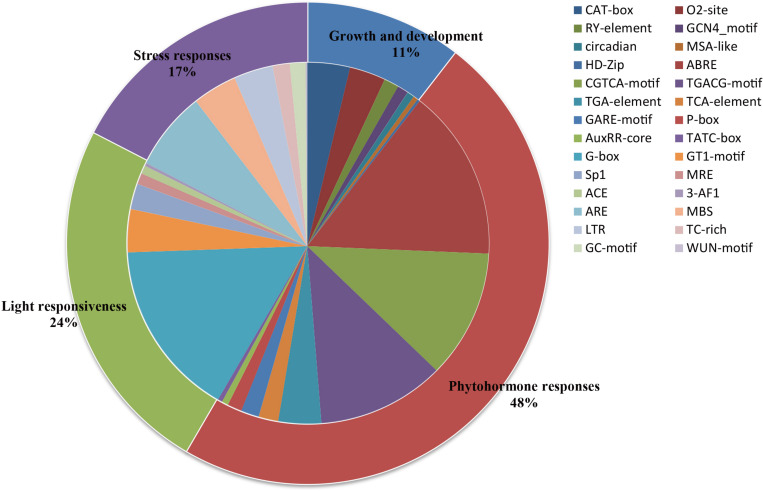
Cis-acting elements of all *PLT* genes in seven Gramineae crops. The ratios of primary categories/cis-elements are displayed by different sizes in the pie charts.

A total of 2,670 cis-elements responsible for growth and development, phytohormone responses, light responsiveness, and stress responses were identified and unevenly distributed on the promoters of all the *PLT* genes (Figure 5D and Supplementary Table S6). The statistics of all cis-elements showed that 48% of them belonged to phytohormone responses and were involved in abscisic acid responsiveness, auxin responsiveness (AuxRR-core and TGA-element), gibberellin responsiveness (GARE-motif, P-box, and TATC-box), MeJA responsiveness (TGACG-motif), and salicylic acid responsiveness (TCA-element) (Figure 6 and Supplementary Table S6). A total of 24% of the elements were light responsiveness elements, including, G-box, GT1-motif, Sp1, MRE, ACE, and 3-AF1 (Figure 6 and Supplementary Table S6). The cis-elements associated with plant growth and development (11%) were CAT-box, responsible for meristem expression; O2, site responsible for zein metabolism regulation; RY, element responsible for seed-specific regulation; GCN4_motif, responsible for endosperm expression; circadian, responsible for circadian control; MSA-like element, responsible for cell cycle regulation; as well as HD-Zip, responsible for the differentiation of the palisade mesophyll cells (Figure 6 and Supplementary Table S6). In addition, ARE, MBS, LTR, TC-rich, GC-motif, and WUN-motif cis-elements were involved in anaerobic induction, drought inducibility, low temperature responsiveness, defense and stress responsiveness, anoxic-specific inducibility, and wound responsiveness, respectively (Figure 6 and Supplementary Table S6).

**FIGURE 7:**
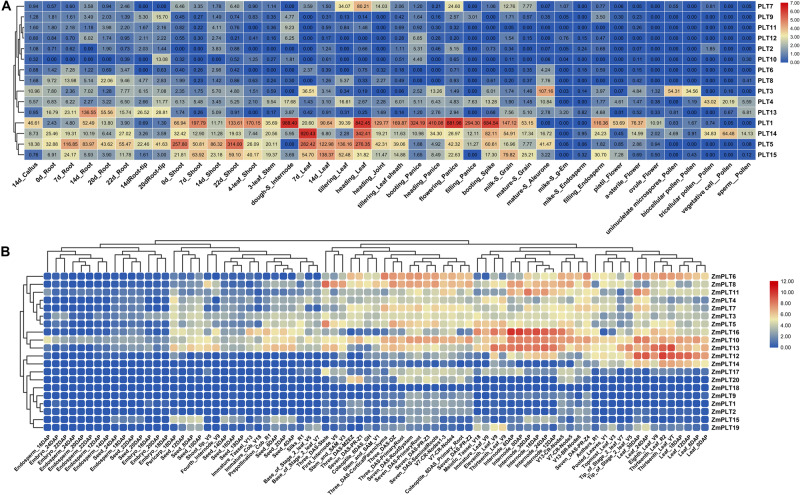
Expression patterns of *PLT* genes in different tissues from rice **(A)** and maize **(B)**. The absolute expression values of rice *PLT* genes were obtained from MBKbase (http://www.mbkbase.org/rice). The absolute expression values of maize *PLT* genes were obtained from maize eFP Browser (http://bar.utoronto.ca/efp_maize/cgi-bin/efpWeb.cgi, data source: Hoopes et al., 2019).

**FIGURE 8:**
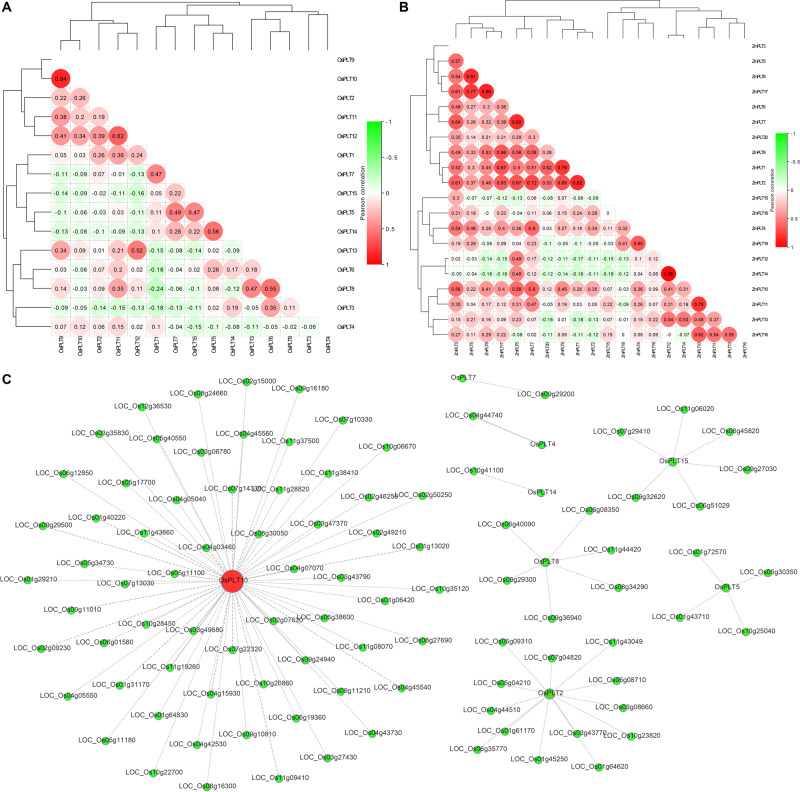
Pearson’s correlation coefficient among *PLT* genes in rice **(A)** and maize **(B)**, respectively. A co-expression network using rice *PLT* genes as query genes was constructed in the Rice Expression Database (http://expression.ic4r.org/) with Pearson’s *r*-value > 0.85 **(C)**.

The expression analyses of the *PLT* genes in rice and maize showed that the *PLT* gene has different expression profiles in various tissues, which indicated that the *PLT* gene may have multiple biological functions and participate in the growth and the development of multiple organs (Figure 7). Several *PLT* genes showed tissue-specific expression, suggesting that they play a role in specific tissues (Figure 7). For instance, *OsPLT13* gene was expressed in a root-specific manner (Figure 7A). Similarly, *OsPLT3* and *OsPLT4* were specifically expressed in pollen at different developmental stages. In order to further explore the functional differentiation of the *PLT* genes in rice and maize, a correlation coefficient between the *PLT* genes was calculated through Pearson coefficient (Figure 8). Most of the correlation coefficients between the *PLT* genes in rice were less than 0.8 (Figure 8A) and the maize species had a similar situation (Figure 8B). However, the correlation coefficients between several *PLT* gene pairs were greater than 0.8, namely, *OsPLT9* and *OsPLT10*, Zm*PLT5* and *ZmPLT8*, *ZmPLT8* and *ZmPLT15*, *ZmPLT6* and *ZmPLT7*, *ZmPLT1* and *ZmPLT2*, as well as *ZmPLT12* and *ZmPLT14* (Figures 8A,B). These results revealed that the *PLT* gene family in rice and maize have undergone significant functional differentiation, but some *PLT* genes may be functionally redundant. Notably, the correlation coefficients of five *PLT* duplicate gene pairs in rice and maize were less than 0.8, namely, *OsPLT11* and *OsPLT12* (0.62), *OsPLT8* and *OsPLT9* (0.14), *ZmPLT7* and *ZmPLT8* (0.32), *ZmPLT12* and *ZmPLT16* (-0), as well as *ZmPLT4* and *ZmPLT6* (0.36), which demonstrated that these *PLT* duplicate gene pairs had obvious subfunctionalization.

### Co-expression Network, GO Annotation, and qRT-PCR of Rice *PLT* Gene Under Heat and Cold Stresses

In this study, a co-expression network using rice *PLT* genes as query genes was constructed on the RED website (Pearson’s *r*-value > 0.85) to find the functional partners of rice *PLT* genes. A total of 96 genes were identified in this co-expression network (Figure 8C and Supplementary Table S6). *OsPLT10* was a hub gene and had 64 partners, while *OsPLT2*, *OsPLT8*, *OsPLT15*, and *OsPLT5* had 13, 6, 6, and 4 partners, respectively (Figure 8C). A GO annotation was performed to analyze the biological processes involved in this co-expression network (Supplementary Table S7). The results showed that the genes in this co-expression network can be annotated to 269 GO terms, suggesting that the co-expression network may be involved in multiple biological processes of rice growth and development (Supplementary Table S7).

**FIGURE 9:**
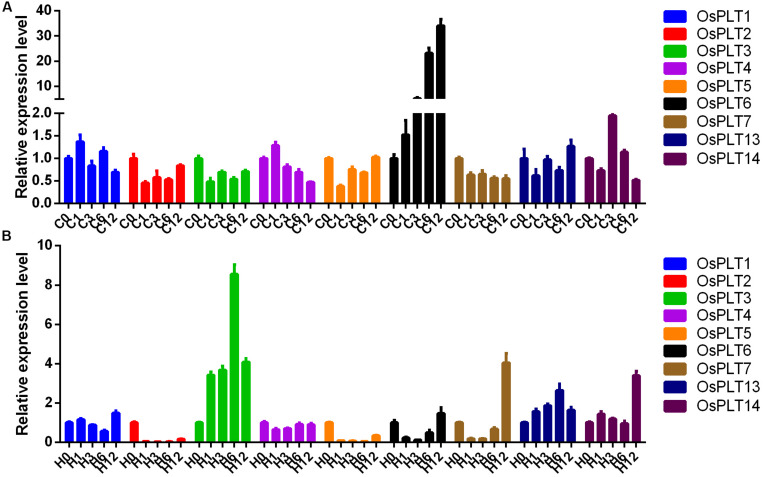
Expression changes of rice PLT genes under cold **(A)** and heat **(B)** stresses by qRT-PCR. The numbers 0, 1, 3, 6, and 12 represent 0, 1, 3, 6, and 12 h after treatments.

The expression changes of nine rice *PLT* genes under cold and heat stresses were detected. The majority of the rice *PLT* genes were down-regulated under heat stress and cold stress (Figure 9). However, the expression level of *OsPLT6* was induced by cold stress. *OsPLT3* and *OsPLT13* had obvious up-regulated expressions under heat stress (Figure 9).

## Discussion

### Gene Duplication and Orthogroup Expansion Promoted the Expansion of the *PLT* Gene Family in Gramineae Crops

Previous gene family studies have demonstrated that gene duplication events play a crucial role in family gene expansion (Büttner, 2007; Van Holle and Van Damme, 2015; Kong et al., 2019b, d). Our earlier studies of MSTs also found that duplication events led to the expansion of the rice STP and ERD gene families (Deng et al., 2019). In this study, we found that gene duplication events also affected the expansion of PTL gene families in some Gramineous crops and ultimately led to differences in the total number of *PLT* genes in these seven tested species. We also found that some OGs in the Gramineous crops had an obvious expansion phenomenon, especially OG1 and OG2. The expansion of OGs had species bias, and the expansion was larger in Sb, Si, and Zm than in Bd, Hv, Or, and Os. On the other hand, the unequal loss of OGs also affected the total number of *PLT* genes in each species. For example, Hv had the least *PLT* gene compared with Bd, Or, Os, Si, Sb, and Zm, which was due to the fact that Hv did not have any *PLT* gene duplication events, that the expansion of OGs was not obvious, and that OG9 was lost. Our results supported the theoretical models of gene family evolution, such that gene families continuously undergo stochastic gain and loss events (Zimmer et al., 1980; Reed and Hughes, 2004). On the other hand, the types and the numbers of *PLT* gene duplication events were not the same in the tested crops, which indicated that the evolution and the expansion patterns of the *PLT* gene family were different in each species. Thus, the Ka/Ks values and the divergence times of the duplicate gene pairs were calculated. All Ka/Ks values of duplicate gene pairs from different types of duplication events were less than 0. Interestingly, the divergence times of the TD duplicate gene pairs (<23 MYa) were later than that of the WGD/SD gene pairs (>24 MYa). This result indicated that different types of gene duplication events were responsible for the expansion of the *PLT* gene family in different evolutionary periods of Gramineous crops, but the driving force and the cause of this phenomenon were still unclear.

### Gene Structure Variations and Different Selection Pressures Promoted the Functional Differentiation of PLT

Previous studies reported that PLT proteins had 12 transmembrane domains and high consistency at the N- and C-termini (Baud et al., 2005; Johnson et al., 2006; Klepek et al., 2010). Our results of the conservative motifs and the transmembrane domain of all PLT protein sequences revealed that the PLTs were very conserved, with 12 transmembrane domains. Internal repeat duplication analysis confirmed that the PLTs protein sequence may originate from an internal repeat duplication of an ancestral six-TMHs unit (Saier, 2000; Johnson et al., 2006). In this study, we found that nine OGs had different purification selection pressures, which provided evolutionary motivation for the functional differentiation of the members of the *PLT* gene family. Significant differences in upstream promoter elements, genomic intron–exon structure, and gene expression among *PLT* genes indeed confirmed that the *PLT* genes were functionally differentiated in rice and maize. The theoretical models of gene duplication proposed that duplicate gene pairs continuously underwent functional differentiation (subfunctionalization), gained new functions (neofunctionalization), or lost original functions (pseudo-genization) after the gene duplication events (Lynch and Force, 2000; He and Zhang, 2005; Innan and Kondrashov, 2010). Gene expression data and co-expression analysis could provide a perspective on the functional fates of duplicate gene pairs (Reinders et al., 2005; Johnson et al., 2006; Kong et al., 2019b). The correlation coefficients of all duplicate gene pairs were less than 0.8, suggesting that these gene pairs had significant functional differentiation. It should be noted that the correlation coefficients between some *PLT* genes in rice and maize are greater than 0.8, which indicates that the *PLT* genes have functional redundancy.

### *PLT* Genes Were Widely Involved in Plant Growth and Development and Stress Responses

The RNA data analysis results from a large number of tissues in rice and maize reflected that the *PLT* genes have a wide range of expression patterns. In this study, we used rice *PLT* genes as the query gene to construct a co-expression network. The GO annotation results of the co-expression network genes showed that multiple GO terms were involved, and *OsPLT10* was the key core gene of the co-expression network. These results indicated that the *PLT* genes have multiple biological functions in plants. Cold and heat stresses frequently severely affect the growth, development, and food yield of crops in many countries (Mittler, 2006; Zhao et al., 2014; Kong et al., 2019b). Previous studies of PLT proteins have focused on transporting monosaccharide types, salt stress response, and drought stress response (Noiraud et al., 2001; Watari et al., 2004; Klepek, 2005; Reinders et al., 2005; Conde et al., 2007, 2011, 2015; Juchaux-Cachau et al., 2007; Dusotoit-Coucaud et al., 2010; Lu et al., 2017). However, few studies have been conducted on the effects of heat or cold stress on the *PLT* genes. In this study, we found that *OsPLT6* was identified as a cold stress-inducible gene, whereas *OsPLT3* and *OsPLT13* were heat stress-inducible genes. These results suggested that the *PLT* genes may also be involved in cold and heat stresses. Although the co-expression network and the qRT-PCR data provided us with clues to the function prediction of rice *PLT* genes and candidate gene screening for genetic breeding, these remain to be experimentally verified in future studies.

## Data Availability Statement

All datasets generated/analyzed for this study are included in the article/Supplementary Material.

## Author Contributions

WK performed all of the experiments, analyzed the data, prepared the figures and the tables, and wrote the manuscript. WK and YL conceived and designed the experiments. TS, CZ, and YQ prepared parts of the figures and the tables. All the authors read and approved the final version of the manuscript.

## Conflict of Interest

The authors declare that the research was conducted in the absence of any commercial or financial relationships that could be construed as a potential conflict of interest.

## Footnotes

1 http://plants.ensembl.org/index.html

2 http://rice.plantbiology.msu.edu

3 http://pfam.xfam.org/

4 http://smart.embl-heidelberg.de/

5 http://pfam.xfam.org/search/sequence

6 https://blast.ncbi.nlm.nih.gov/

7 http://www.cbs.dtu.dk/services/TMHMM/

8 https://swissmodel.expasy.org/

9 http://meme-suite.org/tools/meme

10 http://gsds.cbi.pku.edu.cn/

11 http://www.mbkbase.org/rice

12 http://bar.utoronto.ca/efp_maize/cgi-bin/efpWeb.cgi

13 http://expression.ic4r.org/

## Supplementary Material

FIGURE S1 An internal repeat of *PLT* genes. The proteins themselves are represented by the dark diagonal lines, and the duplicated regions are represented by the above the dark diagonal lines.

FIGURE S2 2D models of 6h7d.1. A and 6n3i.1.A.

FIGURE S3 Sketch map of maize tissues.

TABLE S1 Detailed information on the *PLT* genes in seven tested Gramineous crops.

TABLE S2 Nine orthogroups among the seven tested Gramineous crops.

TABLE S3 Statistics of the four types of orthologs between different Gramineous crops.

TABLE S4 Orthologs list among different Gramineous crops.

TABLE S5 3D structure prediction results of rice PLTs proteins.

TABLE S6 Genes and Pearson’s *r*-values in the co-expression network.

TABLE S7 GO enrichment of genes in the co-expression network.
