# Supplementary figures and images for: Micro-Evolution Analysis Reveals Diverged Patterns of Polyol Transporters in Seven Gramineae Crops
Source: Front Genet. 2020 Jun 19;11:565. doi: 10.3389/fgene.2020.00565 (PMC7317338; doi:10.3389/fgene.2020.00565)

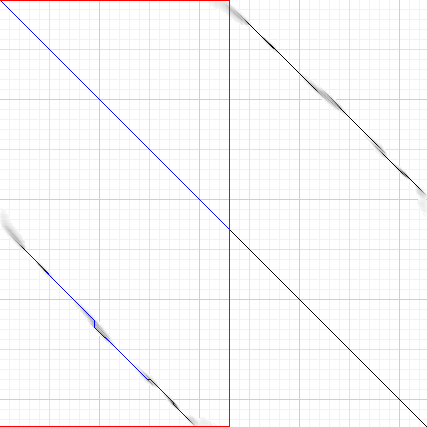

Supplement: FIGURE S1 — An internal repeat of PLT genes. The proteins themselves are represented by the dark diagonal lines, and the duplicated regions are represented by the above the dark diagonal lines. [file Presentation_1.zip › SupMaterial/Figure S1.png]

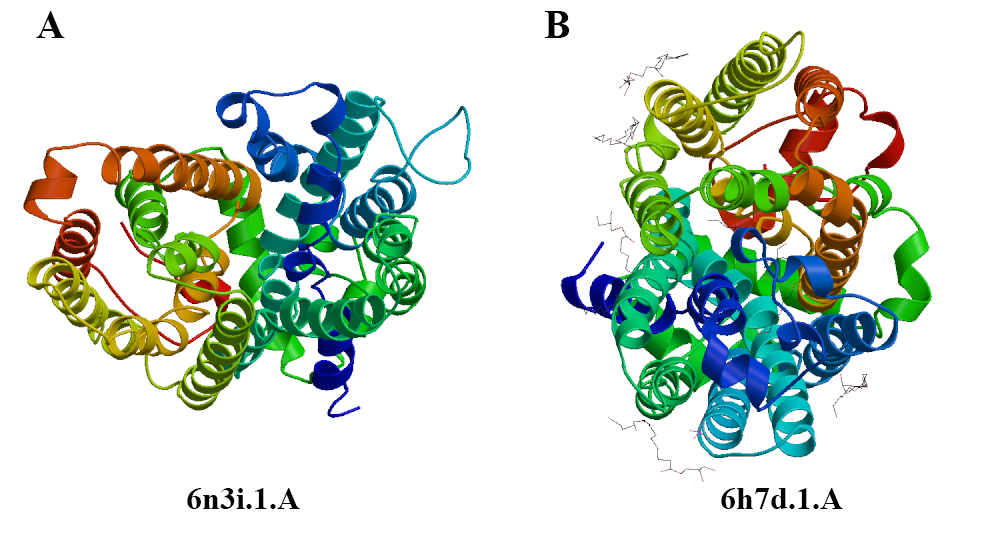

Supplement: FIGURE S1 — An internal repeat of PLT genes. The proteins themselves are represented by the dark diagonal lines, and the duplicated regions are represented by the above the dark diagonal lines. [file Presentation_1.zip › SupMaterial/Figure S2.png]

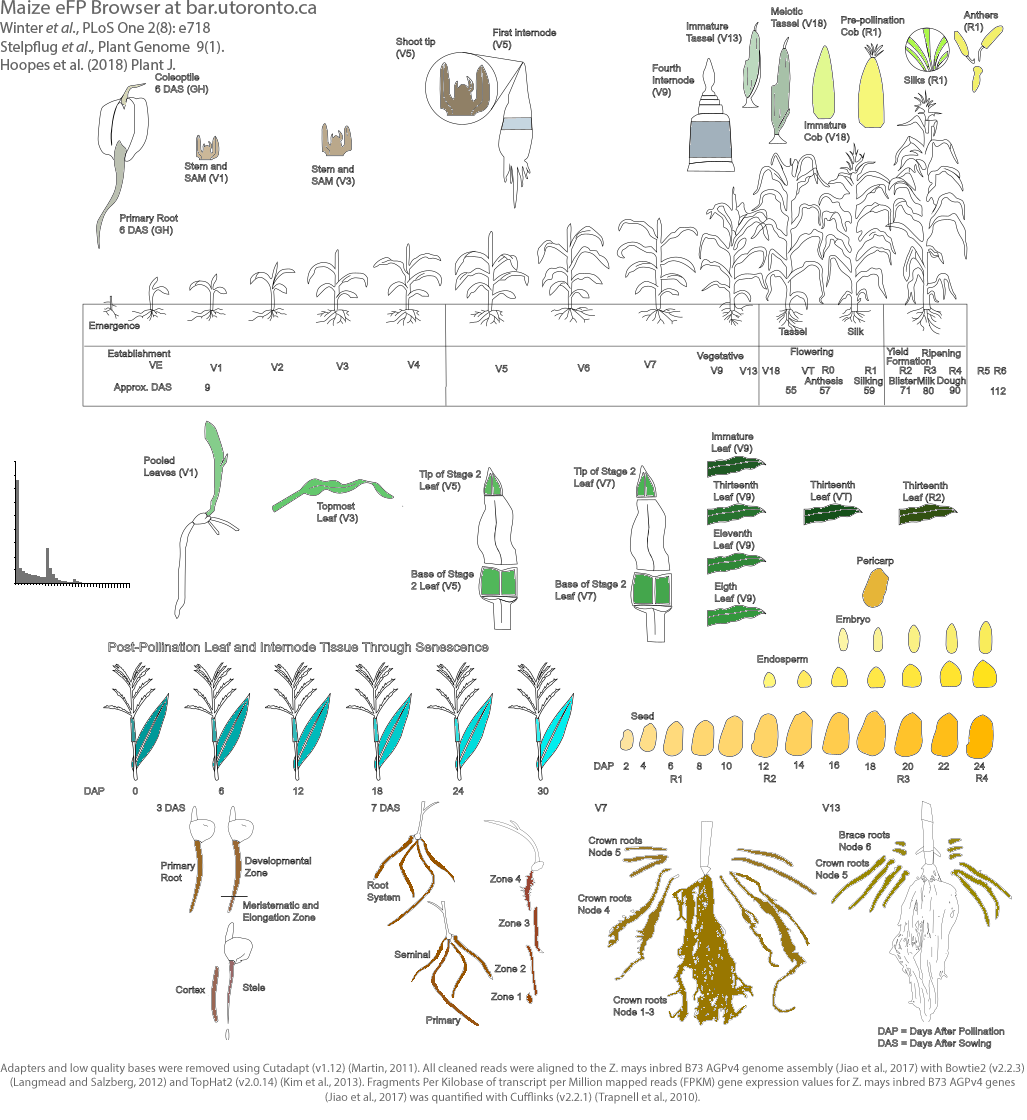

Supplement: FIGURE S1 — An internal repeat of PLT genes. The proteins themselves are represented by the dark diagonal lines, and the duplicated regions are represented by the above the dark diagonal lines. [file Presentation_1.zip › SupMaterial/Figure S3.png]
